# Requirements of Innovative Technologies to Promote Physical Activity Among Senior Citizens: A Systematic Literature Review

**DOI:** 10.1177/00469580251349665

**Published:** 2025-07-07

**Authors:** Alina Napetschnig, Wolfgang Deiters

**Affiliations:** 1 Fachbereich Gesundheitswissenschaften, Bochum, Germany

**Keywords:** requirements, senior citizens, exercise, technical innovations, healthy aging

## Abstract

Technology-based interventions are increasingly recognized as effective tools for promoting physical activity and improving mental health among older adults, particularly in response to the challenges posed by sedentary lifestyles. To ensure these interventions are both effective and sustainable, it is essential to address not only technical requirements but also social and psychological factors, thereby enhancing the quality of life for older populations. The aim of the study is to identify key technical, social and psychological requirements for technologies that promote physical activity among seniors, in order to provide practical recommendations for their development and implementation to enhance acceptance, effectiveness and health outcomes in this target group. A systematic literature search was conducted to identify studies focusing on the requirements of innovative technologies aimed at promoting physical activity in seniors. Databases searched included PubMed, Embase, GeroLit and IEEE Xplore, utilizing targeted keywords to retrieve relevant articles. The identified studies were analyzed through a structured process involving topic identification, code development, categorization and synthesis, culminating in the creation of a comprehensive theoretical framework. A total of 27 relevant studies were included in the analysis. The literature review highlighted a diverse array of technology-based approaches designed to enhance physical activity and quality of life in older adults. Key requirements identified for effective technology adoption included user-friendly interfaces, motivational strategies such as gamification and opportunities for social interaction, as well as accommodations for mobility limitations and robust data protection measures. These requirements span physical, psychological and social domains, underscoring the need for a holistic approach in technology development.

## Introduction

Liu et al^3^ describe that technology-based interventions have emerged as a promising approach to promoting physical activity and improving mental health. Research shows a robust association between physical activity and exercise on the 1 hand and mental and social well-being on the other.^1,2^ Consequently, establishing a consistent regime of physical activity is essential for the individual.^3^ The integration of technological solutions into the daily routines and physical activities of individuals is proving to be feasible. The ubiquitous presence of technology, from smartphones and laptops to gaming consoles and smartwatches, has had a profound impact on diverse areas of life, including daily routines, professional activities and leisure pursuits.^2^ Paradoxically, however, the increasing use of technology has led to a predominantly sedentary lifestyle, which poses a significant public health problem.^3^ This dichotomy emphasizes the need to target technological innovations to promote physical activity and mental health in order to counteract the negative effects of technology use and reap its potential benefits for well-being.^3^

Promoting physical activity and exercise is an essential part of a healthy lifestyle, especially in older age.^4^ In light of demographic changes and an increasingly older population, the development of innovative technologies to promote physical activity among seniors is becoming increasingly important. Technological solutions offer the potential not only to increase motivation for physical activity, but also to address the specific needs of this age group. Both the adaptation of technologies to physical limitations and their integration into everyday life are essential.^5^ Viewing senior citizens as a homogeneous group and automatically equating them with “old people” does not do justice to reality. The functionality and living situation of older people is extremely heterogeneous and individual. While some seniors may be confronted with health restrictions, many others lead active, independent lives full of vitality and commitment.^5^ Chronological age alone says little about physical and mental abilities, interests or lifestyles.^6^ It is therefore important to challenge stereotypes and recognize seniors as a diverse group with different needs, abilities and experiences.

As technology advances, there are more and more innovative devices and applications on the market that are being developed specifically for older people to promote their mobility and physical activity.^4^ From fitness trackers and mobile apps to virtual reality systems and exergaming technologies, the range of possibilities is now enormous. However, despite the growing variety of technologies to promote physical activity among seniors, there are still challenges that need to be overcome to make these technologies effective and user-friendly.

In addition to the basic requirements (such as user-friendliness and safety), this paper also examines physical, psychological and social factors. Promoting physical activity in old age is not just a question of short-term activation, but requires sustainable motivation strategies to ensure continuous use of the technologies.

In this context, the question arises as to which specific requirements innovative technologies must meet in order to motivate seniors to exercise regularly and thereby promote their health and quality of life. These requirements are diverse and include technical as well as social and psychological aspects. This study therefore examines the key requirements for technologies that are developed specifically to promote physical activity among seniors and sheds light on how these technologies can be effectively integrated into everyday life. The aim is to provide an overview of the key criteria that must be taken into account when developing and implementing such technologies in order to achieve sustainable benefits for the target group. Furthermore, practical and theoretically sound recommendations will be given for the development and implementation of technologies that not only increase physical activity, but can also sustainably promote the quality of life and well-being of senior. Both the potential and the challenges of these technologies will be analyzed in order to develop a sound understanding of how they can contribute to the prevention of health problems and the promotion of an active lifestyle in old age. In the course of this, recommendations for future research and development will also be made based on the knowledge gained in order to further increase the acceptance and effectiveness of these technologies.

The research question is:

### How Should (Innovative) Technologies to Promote Physical Activity for Senior Citizens be Designed?

In conclusion, this work aims to emphasize the importance of innovative technologies for promoting physical activity among seniors in the context of demographic development and to contribute to shaping a healthier, more active and socially integrated older society.

## Method

### Methodological Approach Literature Research

This paper presents a comprehensive systematic literature review by identifying, appraising and synthesizing all relevant studies that meet the inclusion criteria. The following 3 research objectives were developed to provide a comprehensive overview of the current studies:

(1) Presentation of innovative technology (groups) that are used to promote physical activity among senior citizens
(2) Comparison of similarities and differences in the literature on requirements for innovative technologies for older people,
(3) Identification of requirements for innovative technologies to promote physical activity for senior citizens for future research activities

This systematic literature search aims to find research related to the requirements of innovative technologies to promote physical activity. The keyword(s) and database(s) to be searched were determined in the review phase. The following databases were used for the search: PubMed, Embase, GeroLit and IEEE Xplore Digital Library.

The following keywords were used to search for related articles: Requirements, innovative, technology, physical activity promotion and senior citizens. The search was carried out using the following term in both English and German:

[“requirements” OR “recommendation”] AND [“innovative” OR “future-oriented” OR “progressive”] AND [“technology” AND “movement” OR “mobilization”] AND [“senior”].

In our review, we considered individuals aged 50 years and older as “older people” or “senior citizens.” This age threshold is consistent with established definitions in gerontological research, which often use 50 years as a lower limit for studies on aging, physical activity and technology adoption among older adults. Spirduso et al^7^ highlight that research on aging and physical activity frequently includes participants aged 50 and above, as this age group begins to experience relevant physiological, psychological and social changes associated with aging.

The literature search, including the analysis process, was conducted in the period 10/2024–02/2025. Some of the publications found in the databases that met the inclusion and exclusion criteria (see Table 1) were identified as duplicates. All relevant studies identified by the database searches were downloaded and saved in the literature management software EndNote, which automatically eliminated the duplicates.

**Table 1.** Inclusion/Exclusion Criteria.

Inclusion criteria	Exclusion criteria
Review articles and research articles that focus on the requirements, development, or implementation of innovative technologies designed to promote physical activity among senior citizens	Review articles and research articles
	Studies that do not relate to the topics requirements, development, or implementation of innovative technologies designed to promote physical activity among senior citizens
Articles in English and German	Articles that were not written in English or German
Articles published between the years 2020 and January 2025	Studies conducted before 2020 or after January 2025 were published
Qualitative, quantitative or mixed-methods research	Studies in which the participants were are 50 years old
Research in which the participants are older are more than 50 years old	The articles did not contain enough information to categorize them
Articles that make further demands on the topic (eg, also technology acceptance models, user experiences, etc.)	

### Methodical Procedure Preparation of Results

The development of a theoretical framework for the investigation of the requirements with regard to technical innovations to promote the mobility of older people is based on a systematic literature review and the synthesis of the relevant content. This approach makes it possible to answer the question comprehensively and to process the results in a structured manner.

1. **Literature search and topic identification**: First, a comprehensive literature search is conducted to identify relevant studies and articles. These sources are carefully evaluated in order to identify the central topics and concepts that are relevant to the study of the mobility of older people.
2. **Initial structuring and evaluation of the content**: Once the relevant topics have been identified, an initial rough structuring of this content takes place. This step makes it possible to evaluate the topics and create an initial tabular list of the central aspects.
3. **Development of preliminary codes**: To better organize the data, preliminary codes are developed. These codes serve as a basis for later categorization and help to group and structure the data.
4. **Categorization and sorting into framework categories**: In a further step, the preliminary codes are grouped and sorted into framework categories.
5. **Synthesis and interpretation of the results**: Finally, the results are synthesized and interpreted to complete the theoretical framework. This framework serves as a basis for answering the research question and provides a comprehensive overview of the mobility of older people.

The coding process follows a systematic qualitative approach – specifically grounded theory – in which key content is first extracted from the data and then transferred into initial codes.

**Figure 1.**
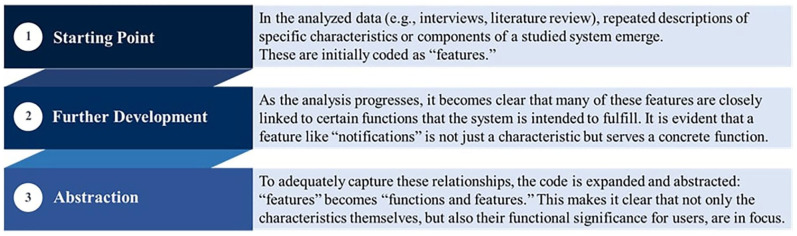
Illustration of the coding and framework development process.

Figure 1 illustrates this process by showing the step-by-step procedure and the example of the “features” category.

In the initial phase of the analysis, recurring descriptions of specific characteristics or components were identified and coded as “features.” As the analysis progressed, however, it became apparent that many of these characteristics were closely linked to particular functions intended to benefit users. To adequately reflect this relationship, the code was expanded and abstracted to “functions and features.” In this way, both the characteristic itself and its functional significance for users are emphasized.

This systematic approach allows the results of the literature research to be processed in a structured manner and presented within a coherent theoretical framework.

We used the PRISMA checklist from EQUATOR to ensure the quality and transparency of our systematic review and to provide comprehensive and traceable reporting of all relevant aspects in accordance with internationally recognized standards.

## Results

### Description of Inclusivism

**Figure 2.**
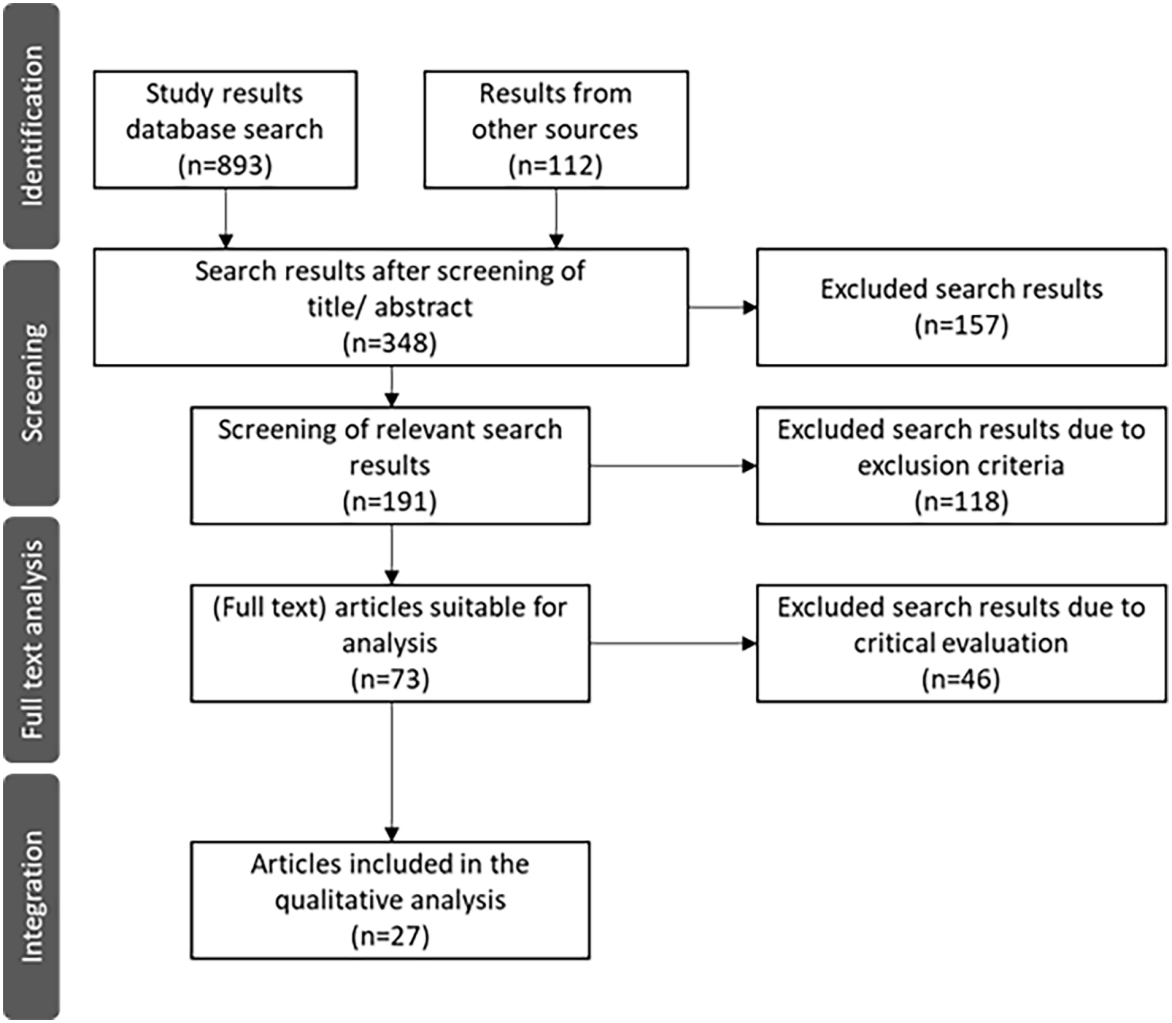
PRISMA (Preferred Reporting Items for Systematic Reviews and Meta-Analyses) flow chart of the literature search process.

The following section describes the results of the literature search. The literature review was conducted using a systematic search strategy that resulted in 893 hits in databases and 112 results from other sources. The selection process was illustrated using a PRISMA flowchart (see Figure 2), which is based on the guidelines of Moher et al.^8^

### Title and Abstract Screening

In the first step, the titles and abstracts of the identified publications were screened. This resulted in 348 publications remaining for further analysis. Of these, 157 studies were excluded as they did not meet the inclusion criteria.

### Full Text Screening

The remaining 191 publications were then screened in full. At this stage, forewords, introductory texts, statements and general position papers were excluded. Each title or summary was evaluated by 2 independent reviewers who could not influence each other. The evaluation was carried out in 2 steps: First, the titles/abstracts were assessed for eligibility, followed by the assessment of the full texts.

### Integration of the Results

To carry out the screening process, EndNote was used to form categories (inclusion or exclusion). This led to 73 results being selected for the full-text analysis. In addition, a further 46 results were excluded as they were thematically unsuitable or only focused on 1 specific topic (eg, data protection) and were therefore not comprehensive enough. A total of 27 results were integrated into the qualitative analysis.

### Description of the Results

The analysis of the research work on the requirements of innovative technologies for promoting physical activity among older people (see Table 1 (Appendix)) shows that different technologies pursue different approaches to improving physical activity and quality of life among older people. The results of the requirements are presented in extracts using individual examples of technology (groups). The requirements were assigned to the corresponding categories derived from the coding process described above. The preliminary codes were “features,” “principles,” “core elements” and “purpose” and became the final codes “functions and features,” “user-oriented design,” “important aspects” and “goals.”

E-health technologies emphasize the importance of automated advice, online resources,^9^ tele-advice and digitally tailored advice. In addition, digital activity recording, digital physical activity coaching and interactive elements such as video vignettes, video demonstrations and video games are crucial for user motivation and engagement. Sensor and communication devices should enable visualization of local dynamics and incorporate contextual information as well as local resources and cultures to increase adoption and effectiveness.^10^ mHealth and eHealth apps rely on synchronization with smartwatches or activity monitors,^11^ behavior change techniques, goal setting, self-monitoring and social rewards. The combination with professional support is seen as particularly effective. Smartphone-based applications emphasize consideration of individual needs, one-on-one support and person-centered technology training.^12^ In addition, motivators such as social connectivity and social contribution should be considered. Feedback and proof of value are crucial to motivate users. The inclusion of health consultations and the promotion of family support are also important. The content description outlined here shows the diversity of technical innovations for promoting physical activity and the requirement criteria that can be derived from them. Overall, the results show that innovative technologies for promoting physical activity among seniors must fulfill a variety of requirements in order to be effective.

Table 2 presents the key findings on innovative technologies to promote physical activity for seniors under different headings. The development of innovative technologies to promote physical activity in seniors requires a variety of functions and features to be effective. These technologies should be characterized by synchronization with activity monitors,^13^ behavior change techniques^13^ and goal setting and self-monitoring,^14^ among others. In addition, the visualization of local dynamics and the inclusion of contextual information is crucial.^12,15^ Automated and tele-counseling provide flexible access to support,^16,17^ while digital activity recording and online resources support users in tracking their activity.^18^

**Table 2.** Key Findings on Innovative Technologies to Promote Physical Activity for Senior Citizens.

Category	Contents	Source
Functions and features	Synchronization with activity monitors	Zhe and Li, 2023
	Behavior change techniques	Zhe and Li, 2023
	Target setting and self-monitoring	Kolb, 2021
	Visualization of local dynamics	Guo et al, 2020
	Inclusion of contextual information	Haynes et al, 2023
	Automated consulting	Fisk et al, 2020
	Tele-consulting	Sayin Kasar and Karaman, 2021
	Customized digital consulting	Fisk et al, 2020
	Digital activity recording	Karanasios et al, 2021
	Online Resources & Videos	Karanasios et al, 2021
	Combination with professional support	Sayin Kasar and Karaman, 2021
	Person-centered technology training	Fisk et al, 2020
	Joint-friendly activities	Zhe and Li, 2023
	Training courses on technology familiarization before use	Costa-Brio, 2024
User-oriented design	Simple user interfaces	Fisk et al, 2020
	Larger fonts	Fisk et al, 2020
	Voice-activated tools	Messaoudi et al, 2022
	Few buttons	Fisk et al, 2020
	Consideration of mobility restrictions	Fisk et al, 2020
	User-friendly interfaces	Busskamp et al, 2022
	Multidimensional approach	Napetschnig et al, 2023
	Suitability for everyday use	Napetschnig, 2024
	Clear communication and instructions	Hua et al, 2024
	Reminders and warnings	Cieślik et al, 2023
	Avoidance of abbreviations and acronyms	Napetschnig, 2024
	Color contrasts	Yap et al, 2022
	Simple gestures	Cieślik et al, 2023
	Intuitive navigation	Napetschnig et al, 2023
	Combination of text and symbols	Yap et al, 2022
Important aspects	Data protection and privacy	Haynes et al, 2023
	Consider motivators	McGarrigle and Todd, 2020
	Feedback on value creation	LaMonica et al, 2021
	Simple functions	Fisk et al, 2020
	No under/overload	Napetschnig, 2024
	Avoid sources of danger (e.g. tripping hazards)	Zhe and Li, 2023
	Check individual suitability before use	Chua et al, 2024
	Integration of feedback, motivation and social interaction elements	Costa-Brio, 2024
Goals	Promotion of health literacy	Haynes et al, 2023
	Support with behavioral changes	Haynes et al, 2023
	Improving medical care	Haleem et al, 2022
	Strengthening patient involvement	Haleem et al, 2022
	Promotion of independence	Chua et al, 2024
	Ensuring health and safety	Zhe and Li, 2023
	Social integration and participation	Chua et al, 2024
	Support for cognitive abilities too	Yap et al, 2022
	Promotion of digitality	Cieślik et al, 2023

A user-oriented design is also of central importance. This includes, for example, simple user interfaces, larger fonts, few buttons and voice-activated tools.^16,19^ Consideration of mobility impairments and the use of user-friendly interfaces are also important.^16,20^ A multidimensional approach and suitability for everyday use ensure that the technologies can be integrated into everyday life.^21,22^

Important aspects include data protection and privacy,^12^ the consideration of motivators^11^ and feedback on value creation.^23^ Technologies should offer simple functions and neither under- nor overburden users.^16,22^ In addition, the integration of feedback, motivation and social interaction elements is crucial.^24^

The objectives of these technologies are diverse: for example, they are intended to promote health literacy, support behavioral change and improve medical care.^12,25^ They also aim to strengthen independence, ensure health and safety and promote social integration and participation.^25^ They should also support cognitive skills and promote digitality.

The development of innovative technologies to promote physical activity in older people requires consideration of a variety of physical, psychological and social requirements (see Table 3) in order to be effective and improve the quality of life of older people.

**Table 3.** Physical, Psychological and Social Requirements of Senior Citizens for Innovative Technologies to Promote Physical Activity.

Requirement area	Requirements	Source
Physical	Consideration of chronic pain	Haynes et al, 2023
	Consideration of physical limitations (e.g. range of motion)	Haynes et al, 2023
	Consideration of fear of falling	Haynes et al, 2023
	User-friendly devices (e.g. large buttons, clear displays)	Napetschnig, 2024
	Ergonomic design to prevent injuries	Ismatullaev et al, 2022
	User-friendly interfaces that are also accessible for people with reduced mobility	Chua et al, 2024
	Customizable screen sizes	Yap et al, 2022
	Clear visual instructions	Cieślik et al, 2023
	Physical stability of the devices	Maresova et al, 2023
	Integration of sensors for motion monitoring	Cieślik et al, 2023
	Support for different mobility levels	Chua et al, 2024
	Easy handling of accessories	Maresova et al, 2023
	Can be used while sitting or standing	Yap et al, 2022
Psychic	Motivation for physical activity through technology-based measures (e.g. games on the Wii)	DNQP, 2020
	Potential negative effects such as cognitive impairment and sleep disorders due to increased screen time	Haynes et al, 2023
	Simple operability and intuitive user interfaces	Napetschnig, 2021
	Motivation through gamification elements	Martinho et al, 2020
	Support in overcoming fears when dealing with technology	Cieślik et al, 2023
	Simple learning curve for new technologies	Chua et al, 2024
	(Positive) feedback on use	Embarak et al, 2021
	Promotion of self-efficacy	Chua et al, 2024
	Adaptation to individual needs	Chua et al, 2024
	Stress reduction through simple operation	Embarak et al, 2021
	Promotion of cognitive skills	Yap et al, 2022
	Support (e.g. with problems)	Chua et al, 2024
	Incentives through competitions or challenges to increase activity	Yap et al, 2022
Social	Online social support to increase effectiveness	Kwan et al, 2020
	Promoting social connectivity through technology (e.g. comparison of step counts)	Haynes et al, 2023
	Role of social networks (family, friends, health professionals) in the use of technology	Haynes et al, 2023
	Opportunities to interact with other users (e.g. group activities)	Napetschnig, 2024
	Involvement and support from family or caregiver	Embarak et al, 2021
	Access to social networks to promote the community	Embarak et al, 2021
	Access to training	Yap et al, 2022
	Cultural sensitivity	Haynes et al, 2023
	Promotion of group activities	Embarak et al, 2021

#### Physical Requirements

Technologies should be designed to address the specific physical needs of older people, including consideration of chronic pain and physical limitations as well as fear of falling.^12^ User-friendly devices with large buttons, clear displays and ergonomic design are crucial to prevent injuries and ensure accessibility for people with reduced mobility.^22,26^ Customizable screen sizes, clear visual instructions, physical stability of devices and integration of sensors to monitor movement are also important to support different levels of mobility.^12^

#### Psychological Requirements

Technologies should increase motivation for physical activity through technology-based measures, such as games on the Wii.^27^ Ease of use and intuitive user interfaces are crucial to avoid cognitive impairment and shorten the learning curve.^28^ Gamification elements can increase motivation, while positive feedback and the promotion of self-efficacy are also important.^29^ In addition, stress reduction through ease of use and the promotion of cognitive skills are key aspects.^12^

#### Social Requirements

Technologies should provide online social support by enabling comparisons of step counts or integrating social networks.^9,12^ Opportunities to interact with other users, such as group activities, are crucial to avoid social isolation.^22^ Involvement and support from family or caregivers and access to social networks can increase the effectiveness of the technologies.^12^ Cultural sensitivity and the promotion of group activities are also important to adapt the technologies to the needs of the users.^12^ Overall, these requirements show that innovative technologies to promote physical activity in seniors require comprehensive consideration of physical, psychological and social aspects in order to be effective and improve the quality of life of older people.

## Discussion

The development of technologies and technology-based interventions to promote physical activity shows an increasing sophistication, which manifests itself in the integration of socio-ecological approaches that combine medical, technological and psychosocial perspectives.^12^ The current state of research on the effectiveness of technological support systems in maintaining and promoting the mobility of older people is still insufficient.^27^ The same applies to IT-supported procedures, where there is also a lack of robust evidence. Nevertheless, technology can be used as a complementary element in mobility-preserving and mobility-promoting interventions.^27^ During implementation, it should be taken into account that technology-based measures can provide a motivational incentive for physical activity for some people in need of care or older people. However, it is essential to carefully weigh up and consider potential negative effects when using technical support systems.

To further contextualize the current evidence gaps and the challenges in implementing technological support systems for mobility among older adults, it is helpful to refer to established theoretical frameworks such as the Technology Acceptance Model (TAM),^30^ UTAUT,^31^ and the WHO’s Healthy Aging framework.^32^ According to TAM and UTAUT, key factors like perceived usefulness and perceived ease of use are central to whether older adults accept and adopt new technologies. For instance, if a mobility technology is perceived as beneficial and easy to use, it is more likely to be integrated into daily routines; conversely, anxiety or low self-efficacy regarding technology can hinder adoption.

The WHO’s Healthy Aging framework further emphasizes that digital solutions should support functional ability and autonomy, aligning with the goal of maintaining mobility and independence in later life. Integrating these models into future research can help identify not only the technical and motivational barriers but also the facilitating conditions and social influences that shape technology acceptance among older adults. This theoretical grounding can guide the development and evaluation of interventions to ensure they are both effective and tailored to the diverse needs of this population.

Further research, particularly on individual technology (groups), is required to comprehensively evaluate the effectiveness and safety of these technologies in the context of promoting mobility among older people.^27^ It should be noted that short-term intervention periods could contribute to high usage rates. However, technology use may be subject to change over time, with initially high engagement levels decreasing in the long term due to the novelty effect.^24^ Based on these findings, it is recommended to integrate e-health interventions into existing guidelines to increase physical activity in older people.^9^ In particular, future e-health interventions should incorporate online social support and automated tracking features (eg, fall detection and activity monitoring) to optimize their effectiveness. Further research is needed to evaluate the relative effectiveness of different e-health strategies and to develop evidence-based recommendations for their implementation.^9^

Haynes et al^12^ add that interventions often implement evidence-based behavior change techniques and persuasive strategies, including activity tracking, exercise reminders, personalization, hedonistic elements, reward systems for goal achievement and mechanisms to promote social connectivity. The authors continue that despite the potential of these technologies, potential negative effects must be considered, such as social isolation due to reduced interpersonal interactions, cognitive impairment and sleep disturbance due to increased screen time and privacy concerns. In addition, activity-based technologies often insufficiently address specific barriers of older people, such as chronic pain, physical limitations or fear of falling.^12^ Research shows that social networks, especially family, friends and health professionals, play a central role in initiating and maintaining the use of technology to promote activity. Peer recommendations and joint participation in age-appropriate online courses were found to be particularly effective. Technology can also serve as a medium for intergenerational exchange and shared interests, for example by comparing step counts or sports performance.^12^ Older users often report ambivalent feelings toward technology, including both enthusiasm and apprehension. Lancu and Lancu^33^ concluded that research shows significant differences in technology literacy between older and younger generations. According to the authors, older adults exhibit lower levels of expertise, limited operational skills (eg, scrolling, clicking) and less experience with operating systems and software compared to younger cohorts. Some studies suggest that some older adults consider themselves “too old” to acquire technological skills. In terms of gender differences in technology anxiety, research provides inconsistent results. While some studies find lower levels of technology anxiety in men, the evidence for a significant gender difference is not clear.

The perception that technology is primarily developed for and by younger, healthy individuals can lead to a stereotype threat that undermines the perceived competence of older users. Haynes et al^12^ describe that features deemed useful by designers may not generate significant added value for older users, the majority of whom are not digital natives. To address these challenges, a paradigm shift is required: Rather than viewing aging as a problem to be mitigated by technology, an approach should be taken that empowers older people to use technology creatively to promote healthy aging and life satisfaction. Central to this is the active involvement of older people from diverse cultural backgrounds as co-designers in the development and evaluation process of technologies. This participatory approach promises to significantly increase the relevance and acceptance of technological interventions in the target group.^12^

The present analysis reveals a significant research gap with regard to innovative and strategic approaches for the adequate integration of digital interventions for seniors. Bridging the digital divide between generations remains a significant challenge. The prevailing technological design paradigm is still primarily based on a supply-oriented approach, in which digital developers assume a universal “one-size-fits-all” principle.^34^ However, according to the authors, this approach neglects the specific needs and abilities of older adults, particularly with regard to physical and cognitive capacities, accessibility requirements, age-related changes and limited fine motor skills.^34^ In addition, previous findings on the (positive) use of technology by seniors are based on short-term interventions. It also highlights the urgent need for guidelines to guide designers, policy makers and community workers in the effective implementation of technology-enabled interventions at the community level. Technology is recognized as a key component in addressing the challenges associated with demographic change.^10^ A key issue among older people is digital exclusion. Some of the studies analyzed excluded participants without internet access or technological devices, which may have influenced technology usage rates and the evaluation of the tools tested. To reduce digital exclusion and inequality, policies to promote digital literacy should be implemented. In addition, technology-based intervention studies should also include older adults without technology experience and with lower levels of education.^24^ Older adults are often perceived as needing support due to functional limitations associated with age and often limited technological competence. The authors describe that it is therefore essential to understand the practices and rhetoric of older people, as these significantly influence the design of technologies for active aging. This discrepancy between technology development and the needs of older users underlines the need for a more inclusive and user-centered design approach. Future research and development should aim to close this gap by systematically integrating the diverse needs and abilities of older adults into the design process.^34^

The findings suggest that the choice of technology can significantly influence the participation of older adults. As the complexity of the skills required increases, the likelihood of meaningful participation decreases.^10^ For example, interventions that rely on smartphone apps or digital displays may present barriers for older adults without appropriate technological familiarity. Many of the interventions examined focus on the use of technologies that older adults are already familiar with. However, as the number of stakeholders involved increases, so does the complexity of the goals, needs and agendas to be considered. In addition, the use of technology in such interventions requires careful consideration of physical accessibility. Consequently, future research and intervention initiatives must comprehensively address these disparities. The focus should not only be on digital literacy and access, but also include the physical infrastructure that is essential for equitable participation.^10^ Costa-Brio et al^24^ add that although some research groups used or adapted commercially developed technologies in their interventions, the involvement of older adults in the technology development process and intervention design remains underestimated. In addition to education, factors such as income, cultural background and technology knowledge also influence the adoption of new technologies among older adults.^24^ LaMonica et al^23^ add that the implementation and acceptance of technology among older adults is influenced by various factors, with frustration identified as a significant barrier leading to a lack of confidence and motivation in using technological solutions. Older adults tend to approach the introduction of new technologies with skepticism about their capabilities, partly due to inadequacies in software and hardware interfaces that make it difficult to access various functionalities. To address these challenges, the authors propose several strategies^23^:

1. *Development of senior-friendly versions of specific technology services*
2. *Integration of a co-design process involving older adults*
3. *Focus on communicating a significant perceived benefit of the technology*

A major obstacle to the effective participation of older adults in the design process is the lack of expertise in product development and programing. To bridge this technology literacy gap, the integration of an educational component into the co-design process is recommended.^23^ Although comprehensive training in complex computer science topics is not practical, teaching basic knowledge of current technologies and their interactions could be of immense value. Co-design partnerships enable the development of useful technologies and the early reduction of barriers already in the design phase.^23^ This proactive approach addresses potential problems that can arise from low technology literacy and restrictive privacy settings before a product is launched. In addition, feedback loops can be implemented to help older adults better understand their data and how it is used to predict their health needs. This integrative approach promises to increase the acceptance and effective use of technology among older adults by incorporating their specific needs, abilities and concerns into the development process from the outset.^23^ The authors Costa-Brio et al^24^ add that technology-based systems are considered promising tools to promote and improve physical functioning of older adults in the home environment. However, according to the authors, the lack of familiarity of many older people with technology can lead to difficulties and affect the acceptance and adherence of digital interventions.

Stara et al^5^ agree with the findings and add that the acceptance and success of technological solutions for older adults is dependent on several critical factors. A key aspect is the user’s perception of privacy, with data security and confidentiality identified as priorities for acceptance, particularly of smart home systems.^5^ For successful aging, the active participation of older adults in social activities and the establishment of a sense of belonging as an integral part of society is of significant importance. However, according to Stara et al,^5^ various factors can impair the effective use of digital technologies in this age group: Firstly, the authors count low digital literacy: as many older adults have not grown up with digital technologies, they have to learn and adapt these skills later in life, which is a potential barrier. Secondly, the authors cite cognitive impairments: A certain degree of cognitive impairment can limit the ability to use technology effectively.

To address these challenges, it is recommended to follow a user-centered design approach in technology development.^5^ This approach aims to counteract possible limitations and optimize the user experience by taking into account the specific needs and abilities of the target group. The implementation of these principles promises to increase the acceptance and effective use of technology among older adults by reducing barriers and improving the user experience. This holistic approach takes into account both the technological and psychosocial aspects of technology use in old age and thus contributes to a more successful and self-determined aging process.^5^ When designing and implementing health-promoting technologies for physical activity promotion for older adults, various practical aspects must be taken into account, especially if the target group has limited experience with technological solutions.^11^ A key factor is the “digital divide,” which may be driven by socio-economic, age, geographical and cultural factors. Effective technology-based interventions need to match the lifestyles and expectations of older people and provide customizable solutions that take into account personal preferences and abilities.^11^ The authors add that intrinsic factors such as a sense of control, a desire for independence and perceived needs or safety requirements play a decisive role in the motivation to use technology. Extrinsic factors include ease of use, technological feedback and cost aspects. In order to promote sustainable acceptance, it is essential to clearly communicate the positive effects of the technology, particularly in terms of promoting independence. The technologies must be perceived as reliable and effective to ensure long-term use.^11^ When introducing new technologies, such as apps, it should be noted that older people may face a steeper learning curve. It is therefore essential to establish adequate support structures to help users familiarize themselves with the technology and overcome any barriers. These multifactorial considerations emphasize the need for a holistic, user-centered approach to the development and implementation of health-promoting interventions using technology for older adults.^11^

The Commission of Experts for Research and Innovation (EFI)^35^ confirms the need for a user-centered approach: The integration of age-tech hubs into the innovation landscape offers promising opportunities to promote technological competence among older people.^35^ These hubs act as interfaces between the areas of mobility, housing, health, care and financial services, enabling a holistic approach to technology adoption in old age. In addition, physical and virtual learning and experimentation spaces can help seniors gain practical experience with new technologies, which in turn provides valuable insights for the requirements analysis.^35^

Support services, such as those initiated by consumer advice centers, can play a key role in promoting design knowledge and improving understanding of the specific needs of older people. These initiatives can act as a link between innovators and end users and thus drive the development of target group-oriented solutions. In order to strengthen the innovative power of individuals, comprehensive promotion of innovative capacity through educational measures from early childhood education to lifelong learning is required.^35^ Employers should also create scope and develop management cultures that encourage experimentation and innovation. The implementation of experimentation clauses and the establishment of innovation departments and technology scouts in the social economy can support this process. Institutionalized evaluation structures, such as real-world laboratories, could help to expand knowledge regarding the use and acceptance of technology. Increased thematic cooperation, supported by digitalization, should be sought here. The promotion of social and technical innovations to support independent living in old age through public funding should be intensified and better coordinated according to EFI.^35^ Continuous updating of the aid catalogs is necessary in order to realize the potential of incremental and radical innovations for prevention and maintaining quality of life. Funding should cover both the provision of digital technologies and target group-oriented advice, education and support services. In view of the unequal distribution of access to and use of technical and social innovations within the group of older people, measures are needed to guarantee Internet access in all forms of housing for older people. The areas of mobility, neighborhoods and housing are central aspects of quality of life in old age. The potential for barrier-free living space design as well as for assistance systems (Ambient Assisted Living, AAL) and home automation (Smart Home) is considerable. Innovative mobility solutions such as mobility apps, smart wheelchairs and exoskeletons are becoming increasingly important.^35^ Support services such as training, technical support and digital help platforms can positively influence both the invention and diffusion of technical and social innovations. The institutional promotion of community health nurses and case/care managers can act as a driver for technical and social innovations in the health and care sector.^35^ An adaptation of care and therapy service catalogs is necessary to enable the spread and use of digital innovations in practice. Higher individual care budgets could stimulate demand for social and technical innovations. Promoting the ability to innovate in the social economy through experimentation spaces, experimentation clauses and qualification offers can strengthen both the power of invention and the diffusion of social and technical innovations. Finally, assistive technologies, service robotics, smart home applications, serious games, care and health apps and e-health applications open up new possibilities for supporting people in need of care and care staff. These innovations offer potential for prevention, participation, social contact and mobility as well as cognitive, emotional and physical activation.^35^

## Limitations

This systematic review is subject to several limitations that should be considered when interpreting the findings. First, only published studies were included in the analysis, which may have resulted in publication bias, as studies with non-significant or negative results are less likely to be published and thus may be underrepresented. Second, the review was limited to studies published in English and German, potentially excluding relevant research published in other languages and thereby restricting the comprehensiveness of the evidence base.

Another limitation concerns the heterogeneity among the included studies, both in terms of intervention types, technological platforms and outcome measures. This diversity complicates direct comparisons and synthesis of results, and may limit the generalizability of the conclusions.

Most of the included studies were conducted in Western, industrialized countries, with limited representation from developing regions and diverse ethnic or socio-cultural backgrounds. As a result, the recommendations derived from this review may not be fully generalizable to all global populations. Future research should aim to include a broader range of geographical and cultural contexts and report participant backgrounds in more detail to enhance the applicability and inclusiveness of findings.

A further limitation of this review is that economic factors such as affordability, as well as aspects of education and digital literacy, were not systematically assessed across the included studies. The accessibility and sustained use of technology-based interventions are strongly influenced by users’ financial resources, educational background and digital skills. The lack of detailed reporting on these factors in many studies limits the generalizability of our findings, particularly regarding the real-world applicability of technological solutions for diverse older populations. Future research should systematically examine and report on economic and digital literacy barriers to ensure that recommendations for technology adoption are inclusive and actionable for all segments of the older adult population.

Given the rapid pace of technological development, some of the included studies may already be outdated and the findings may not fully reflect the most recent innovations or current user preferences. In addition, although the review aimed to address requirements for seniors, many studies did not differentiate between genders or other relevant subgroups, limiting the specificity of the conclusions for this target population.

Finally, most studies focused on short-term outcomes, with limited evidence regarding the long-term adoption, sustained use, and effectiveness of the technologies in promoting physical activity among older adults. These limitations highlight the need for future research to address these gaps by including a broader range of languages, systematically assessing study quality, focusing on long-term outcomes and considering subgroup-specific requirements.

## Conclusion

Challenges in evaluating technology use include the lack of consensus on technology-related concepts and the lack of validated measurement tools. Nevertheless, the use of technology to provide home exercise for people aged 65 years and older can be considered a promising and effective alternative to increase physical activity and improve physical functioning.

Future research should include individual characteristics in the analysis of technology use and consider more heterogeneous samples in terms of educational level, socioeconomic characteristics and technology experience. With appropriate support, older adults are quite capable of using technology autonomously. Further efforts are needed to include older adults in the design and development of technologies to improve physical functioning. Future interventions should include a more diverse participant sample and longer follow-up periods to ensure the sustainability and effectiveness of technology use over time.

## Supplemental Material

sj-pdf-1-inq-10.1177_00469580251349665 – Supplemental material for Requirements of Innovative Technologies to Promote Physical Activity Among Senior Citizens: A Systematic Literature Review Supplemental material, sj-pdf-1-inq-10.1177_00469580251349665 for Requirements of Innovative Technologies to Promote Physical Activity Among Senior Citizens: A Systematic Literature Review by Alina Napetschnig and Wolfgang Deiters in INQUIRY: The Journal of Health Care Organization, Provision, and Financing

## Appendix

**Table 1.** Contents of Individual Research Studies on the Requirements of Innovative Technologies to Promote Physical Activity Among Senior Citizens.

No.	Authors	Brief description contents
1	Kwan et al, 2020	This review aimed to evaluate the effectiveness of e-health interventions to promote physical activity in older people by analyzing randomized controlled trials. The results showed that e-health interventions can significantly increase physical activity time, energy expenditure and number of steps, with effect sizes varying from small to large. The listed requirements for e-health technologies include automated counseling, online resources, tele-counseling, digitally tailored counseling, digital activity recording, digital physical activity coaching, video vignettes, video demonstrations, and video games.
2	Chang et al, 2024	This systematic review examines the role of technology in community-based interventions to promote active ageing. It analyzes 13 studies with a total of 14 interventions to understand the challenges and opportunities of integrating digital technologies. The results show that it is important to overcome the challenges in community-based interventions and use technology to enhance the desired effects. The authors cite the following technology requirements, among others: Visualization of local dynamics.
		Inclusion of contextual information and tapping into local resources and cultures. It highlights the lack of innovative approaches to effectively incorporate digital interventions into community-based programs, underscoring the need for guidelines for designers, policymakers, and community personnel.
3	McGarrigle and Todd, 2020	Older people are at increased risk of negative health events due to reduced physical activity, exacerbated by the COVID-19 pandemic. Mobile health (mHealth) and eHealth technologies could help older people stay physically active during physical distancing. A review that analyzed systematic reviews found that there is evidence that mHealth and eHealth interventions can increase physical activity in older adults in the short term. Successful interventions include self-monitoring, incorporation of theories and behavior change techniques, and social and professional support. The authors describe that the following requirements, among others, should be met: Synchronization with smartwatches/activity monitors, behavior change techniques, goal setting, instructions for use, self-monitoring, social rewards, and combining the app with professional support.
4	Haynes et al, 2023	Digital technologies offer new opportunities to promote physical activity as an essential component of healthy ageing, but their potential has not yet been fully realized. A study of 17 older adults who use digital technologies to support their physical activity shows that these technologies are perceived as motivators and supporters, despite the challenges associated with ageing and the COVID-19 pandemic. The study identified four key lessons and recommends leveraging trusted social and health relationships to encourage the use of digital technologies to promote physical activity among older people. The authors mentioned the following requirements: Consider individual needs of users, One-to-one support, Person-centered technology training, Consider motivators of older people, such as social connectivity or contributing to society, Feedback: technology needs to demonstrate value to motivate people to use it, Health consultations with GPs, physiotherapists etc., Develop strategies to promote the potential of family support/peer-to-peer engagement and provide IT help (e.g. through family/friends).
5	Stara et al, 2022	This study develops a protocol for a feasibility study that investigates the acceptability, usability and efficiency of the SAVE system, a technology-based system to support older people to live independently. The SAVE system includes environmental sensors, smartwatches, smartphones and a web application and is being tested in the homes of older adults in Romania, Italy and Hungary. The study involves a total of 165 participants, including older people, professional and informal caregivers and decision makers in the field of care services, and uses a mixed methodology with standardized tests and questionnaires. The authors mentioned the following requirements: Sense of confidence and security, contribute to independence in living, increase control of (daily) tasks, physical safety and social communication and demonstrable improvement in physical safety and social communication.
6	Kolb, 2021	The ADCES7 Self-Care Behaviors Framework is a comprehensive approach to self-management of diabetes and other related conditions based on seven key behaviors, including healthy eating, physical activity and health monitoring13. This model promotes person-centered healthcare collaboration and aims to achieve better clinical outcomes and improved quality of life through behavior change24. Integration into the digital and dynamic healthcare sector allows the model to evolve and expand its application to other chronic conditions, with technological advances playing an important role
7	Guo et al, 2020	The concept of “Digital Earth” has evolved over the last 20 years and has become critical to the collection, processing, analysis and use of large global data sets about the Earth. The main aspects of Digital Earth include technologies such as remote sensing, artificial intelligence, the Internet of Things and social media, which are used to address global challenges such as climate change and disaster response. The book provides a comprehensive introduction to the science and technology of Digital Earth, including its applications in areas such as digital cities and cultural heritage, as well as a discussion of future trends and challenges.
8	Fisk et al, 2020	he second edition of Designing for Older Adults: Principles and Creative Human Factors Approaches provides updated guidelines and tutorials for designing technologies that are accessible to older adults. The new edition includes new chapters on topics such as transportation and aging in place, as well as a tutorials section that provides practical guidance for research and usability studies with older adults. The authors emphasize the importance of human factors design in developing technologies that are both effective and safe for older users. The book provides specific guidance applicable to current and future technologies, including the design of multimedia products for older adults.
9	Sayin Kasar and Karaman, 2021	The COVID-19 pandemic has had an unprecedented impact globally, particularly on older people, leading to a deterioration in social inclusion and wellbeing. A comprehensive literature review found that technology could play an important role in reducing social isolation and loneliness in older people by using digital opportunities to promote social contact. Recommendations include the use of technology, cognitive behavioral therapies and individual interventions to improve the quality of life of older people during the pandemic.
10	Karanasios et al, 2021	This thesis highlights the role of activity theory in addressing theoretical and practical challenges posed by the increasing importance of digital technologies in human activities. Activity theory is used as a framework to study and generate new insights into digital technologies such as social media, smartphones, blockchain, artificial intelligence and algorithmic decision making. The work aims to promote future research that will deepen the impact of digital technologies on human activities and advance the development of activity theory in the context of modern technologies.
11	Costa-Brio, 2024	Technology-based systems are considered promising tools to promote physical function in older people at home, but unfamiliarity and uncertainty about the technology can present challenges. This review shows that home-based physical activity interventions mediated by technology can achieve positive health-related outcomes in older adults, with technology use rated as satisfactory in most studies. When older people receive appropriate support, they are able to use technology independently, which promotes their autonomy and health.
12	Messaoudi et al, 2022	Visually impaired people face significant challenges in getting around as they have difficulty moving safely and protecting themselves from moving and stationary objects, which limits their mobility and confidence. The development of assistive technologies has become an important area of research to improve the mobility and independence of visually impaired people. Modern assistive technologies such as artificial intelligence-powered navigation systems, smart canes with ultrasonic sensors and other innovative devices offer new possibilities to support mobility in indoor and outdoor environments and are discussed in detail in the study.
13	Busskamp et al, 2022	Promoting physical activity and social interaction has a positive impact on the health and quality of life of older people. The expertise provides a scientific overview of effective measures to promote social participation and physical activity among older people, including quality-assured practice projects and recommendations for action. Although the text does not mention specific technologies, digital platforms and technologies could play an important role in promoting social interactions and supporting physical activity, especially in combination with physical activities and social projects.
14	Napetschnig et al, 2023	Virtual reality (VR) is becoming increasingly important for older adults as it can improve their quality of life through immersive content that enhances cognitive and motor skills and enriches daily life. To ensure a positive impact, it is crucial to develop VR experiences that are tailored to users’ needs and preferences. The study aims to develop a set of quality criteria and guidelines for user-centered VR applications for older adults, which emerged through an iterative process of literature review, framework analysis and expert workshops.
15	Napetschnig, 2024	Virtual reality (VR) can be supportive for seniors by promoting cognitive and motor skills and having a positive impact on daily life, which can be achieved through user-oriented development. The research project has developed a quality criteria core set for VR applications suitable for senior citizens, which was created through literature research, framework analysis and expert workshops. The finalized quality criteria core set includes various categories such as quality assurance, data protection and interaction and serves as a basis for future VR developments that are adapted to the needs of seniors.
16	LaMonica et al, 2021	The aging population requires specially designed digital tools tailored to their unique needs, as age-related changes in cognition, vision, hearing and perception can present barriers to technology use. This study aims to understand older adults’ use of health information technologies (HITs) and identify potential barriers and facilitators to facilitate implementation and utilization. The results show that personalization of content, access to trusted information, and integration with existing health practices promote the adoption of HITs, while privacy concerns are a major barrier.
17	Haleem et al, 2022	The Fourth Industrial Revolution will significantly impact the healthcare sector by accelerating medical advances and making them more effective, leading to more consistent availability of healthcare services. Medical 4.0, part of this revolution, utilizes emerging technologies such as mobile and cloud computing and the Internet of Things (IoT) to create a highly connected healthcare environment where patient data is collected and used electronically . This digital transformation is shifting the focus from doctor-centric to patient-centric care, enabling better customization to patients’ needs and improving healthcare in both developed and less developed countries.
18	Ismatullaev et al, 2022	The growing elderly population in many developed countries urgently requires products and services that improve their wellbeing, with numerous assistive technologies being developed to support older people both at home and outdoors. However, the application of human factors is often neglected in the development of these technologies, which can lead to design flaws that do not adequately address the abilities and needs of older people. By identifying human factors and ergonomic issues, designs can be developed that are better suited to the needs of older people, increasing their wellbeing and reducing potential errors in technology use
19	DNQP, 2020	The expert standard “Maintaining and promoting mobility in care” was updated in 2020 to ensure and further develop quality in care, with a focus on the mobility of people in need of care. This standard includes process criteria that support care professionals in the assessment, planning and implementation of measures to maintain and promote mobility. For example, it is discussed that the integration of technologies such as wearables or digital platforms for monitoring and promoting mobility could play a role in the future in order to increase the effectiveness of measures.
20	Napetschnig, 2021	The use of virtual worlds (VR) shows promising results in the relief of chronic pain and could serve as an alternative therapy medium to conventional treatments. VR applications have great potential in rehabilitation and offer an innovative way to treat pain without medication. However, to fully exploit this potential, specific technological requirements are needed, such as high immersion quality and user-friendly interfaces to ensure effective pain relief.
21	Martinho et al, 2020	The global population is ageing rapidly, increasing the need for personalized healthcare services to support active ageing, where technologies such as gamification can play an important role. Gamification techniques have been shown to be beneficial in improving the wellbeing of older people by promoting physical, cognitive, social and emotional aspects, using technologies such as self-management systems, wearable devices and wearables. To ensure the success of gamification in elderly care, traditional healthcare services need to integrate and adapt these techniques to the individual needs and abilities of older people, which is a challenge.
22	Zhe and Li, 2023	The demand for rehabilitation training for the elderly in China is increasing due to the aging population and the increasing number of patients with paralysis due to strokes and other diseases, and the monotony of exercises often leads to boredom and slower recovery. This study uses methods such as the affinity diagram method, the Kano model and the Analytic Hierarchy Process (AHP) to analyze the needs of users of somatosensory rehabilitation systems and identify critical design requirements to optimize the rehabilitation experience. The results show that physiological rehabilitation and professional guidance are the most important needs, leading to the development of modules such as a somatosensory rehabilitation game and a virtual assistant robot.
23	Chua et al, 2024	Mobility is crucial for older people to live independently, however many encounter challenges that can be addressed by technology-enabled mobility solutions. These solutions include a variety of technologies that aim to support and improve mobility for older people, however research gaps still exist to fully understand the current state of research. By analyzing the research results, important insights can be gained into the factors that influence the acceptance and use of these technologies, which is important for future research directions.
24	Cieślik et al, 2023	Technological advances make it possible to support rehabilitation for older people through virtual reality (VR), exergaming, serious gaming, wearables and telerehabilitation to improve balance and functional mobility. This study aims to compare the effectiveness of these interventions, as no comprehensive comparisons have been conducted to date, and shows that exergaming with motion capture technology is particularly effective. To fully exploit the effectiveness of these technologies, specific technological requirements tailored to the needs of older people are needed.
25	Embarak et al, 2021	Social integration through communication with family and friends is particularly important for older people as it promotes a sense of appreciation and recognition, which can be supported by online social communities. However, older people are often reluctant to use new technologies, which is why researchers are implementing purpose-built social media applications with user-friendly interfaces in simple devices. This study uses a systematic literature review to analyze different user devices used to promote social connections among older people.
26	Yap et al, 2022	The ageing population and increased life expectancy are widespread social changes in which technology can play an important role in improving the daily lives of older people and maintaining their health. Despite the benefits of technology, older people are slower to adopt new technologies than younger adults due to various influencing factors. This study conducts a systematic literature review to identify the various influencing factors for older people’s technology use and classifies them into seven categories, including technological, psychological, and social factors, to develop a conceptual model for older people’s technology use.
27	Maresova et al, 2023	The health and physical fitness of older people are in focus, as reduced mobility is an early sign of declining fitness and increases the risk of falls, injury and hospitalization. This study identifies various factors that influence mobility in older people, including environmental, physical, cognitive, psychosocial and technological factors, and emphasizes the need for further research in these areas. To effectively support the mobility of older people, comprehensive solutions are needed that take into account the interactions between these factors and integrate innovative technologies such as smart home technologies and assistive technologies, while also exploring financial aspects.
